# Characteristics and electrochemical performances of silicon/carbon nanofiber/graphene composite films as anode materials for binder-free lithium-ion batteries

**DOI:** 10.1038/s41598-020-79205-1

**Published:** 2021-01-14

**Authors:** Ruye Cong, Jin-Yeong Choi, Ju-Beom Song, Minsang Jo, Hochun Lee, Chang-Seop Lee

**Affiliations:** 1 Department of Chemistry, Keimyung University, Daegu, 42601 South Korea; 2 Department of Chemical Education, Kyungpook National University, Daegu, 41566 South Korea; 3 Department of Energy Science and Engineering, DGIST, Daegu, 42988 South Korea

**Keywords:** Chemistry, Energy science and technology, Materials science

## Abstract

We report the interfacial study of a silicon/carbon nanofiber/graphene composite as a potentially high-performance anode for rechargeable lithium-ion batteries (LIBs). Silicon nanoparticle (Si)/carbon nanofiber (CNF)/reduced graphene oxide (rGO) composite films were prepared by simple physical filtration and an environmentally-friendly thermal reduction treatment. The films were used as high-performance anode materials for self-supporting, binder-free LIBs. Reducing graphene oxide improves the electron conductivity and adjusts to the volume change during repeated charge/discharge processes. CNFs can help maintain the structural stability and prevent the peeling off of silicon nanoparticles from the electrodes. When the fabricated Si/CNF/rGO composites were used as anodes of LIBs, the initial specific capacity was measured to be 1894.54 mAh/g at a current density of 0.1 A/g. After 100 cycles, the reversible specific capacity was maintained at 964.68 mAh/g, and the coulombic efficiency could reach 93.8% at the same current density. The Si/CNF/rGO composite electrode exhibited a higher specific capacity and cycle stability than an Si/rGO composite electrode. The Si/CNF/rGO composite films can effectively accommodate and buffer changes in the volume of silicon nanoparticles, form a stable solid–electrolyte interface, improve the conductivity of the electrode, and provide a fast and efficient channel for electron and ion transport.

## Introduction

Currently, rechargeable lithium-ion batteries (LIB) are commonly being used in portable electronic devices, power tools, electronic vehicles (EVs), and medical devices, and they are considered to be ideal energy sources with high energy density, cycle stability, and environmental friendliness. To satisfy the growing demand for higher energy densities and longer cycle life, researchers are continually trying to improve the electrochemical performances of LIB electrodes and design synthetic nano-structures to improve the energy density and rate performance of LIBs^1–3^.

Silicon is a promising anode material for LIBs with a high theoretical capacity (4100 mAh/g, Li3, 75 Si, the most lithiated alloy in the surrounding environment), low working potential, and low price^4^. However, when silicon is used as a negative electrode material, silicon particles undergo significant volume expansion and contraction (approximately 300%) in the processes of lithiation and delithiation, respectively. The large volume expansion will cause the pulverization of the materials and loss of electron contact between the Si particles, thereby reducing the contact area with the current collector, resulting in reduced battery efficiency, reduced cycle life, and sudden breakage of the battery cells^5^. Additionally, the commonly used electrolyte a solid–electrolyte interface (SEI) on the surface of the silicon particles at a potential below 1 V. During volume change, the SEI may rupture to expose bare silicon particles, and consequently an increasing number of SEIs are formed on the exposed silicon surface. The exposed silicon particle surface continuously increases the total layer thickness, rapidly filling the electrode holes, thereby preventing the diffusion of lithium ions into the silicon electrode, eventually reducing the battery capacity. Additionally, because the poor conductivity of silicon does not help obtain an excellent rate performance, thus limiting the practical applications of silicon in LIBs^6–11^.

To solve the serious problems due to volume expansion, researchers have developed various methods to minimize these undesired reactions between the electrode and electrolyte by avoiding direct contact between the silicon surface and electrolyte to the maximum possible extent and striving to improve the cycle performance and rate capabilities^12^. In previous research reports, silicon nano-composites, thin films, and nano-structured silicon (including Si nano-tubes^13^, Si nano-wires^14^, Si nano-spheres^15^, nano-arrays^16^, nano-porous structures and their composites with carbon materials and conductive polymers^17^) were studied, as these materials have high resistance to the structural fracture caused by changes in the silicon volume, thereby reducing the material crushing and improving the cycle performance. However, nano-wires and nano-tubes, which are expensive and difficult to scale up, have low mass fractions of active ingredients in electrodes, thereby resulting in limited power storage capacity. Therefore, by dispersing silicon into other materials, a composite material that has a stable structure and can buffer volume changes is obtained to improve the electrical conductivity and cycle stability^18,19^. Carbon-based materials are rapidly being recognized as high-efficiency coating materials for preparing LIBs owing to their high conductivity, environmental friendliness, and excellent physical and chemical stabilities^20,21^. The coating of carbon-based materials (including amorphous carbon, graphite, carbon nano-tubes, carbon nanofibers, and fullerenes.) improves the electrochemical performances of silicon materials in LIBs. It also improves the electrical conductivity, and buffers the volume change of the silicon particles to form a stable SEI.

Graphene is an innovative, two-dimensional carbon nano-material with excellent electron and ion transport characteristics, high thermal stability, high mechanical flexibility, high lithium storage capacity, and high specific surface area^22,23^. These properties provide great advantages for improving the reversible capacities of composite materials. However, the theoretical capacity of graphene is only 372 mAh/g. Previous experiments showed that many active materials grown on graphene or wrapped in graphene sheets, such as carbon–silicon core–shell nano-wires, Si/C nano-spheres, Si/C microspheres grown on graphite microspheres, and composite materials such as graphene and carbon nanotube-coated silicon, can adapt to this volume change, improve the electronic and ionic conductivities of Si, improve the reversible capacity effectively, and improve the cycle and rate performances of LIBs^24,25^. However, graphene can be stacked very easily in the process of manufacturing an electrode, and the mutual penetration between graphene layers is insufficient, and thus the diffusion distance of Li^+^ through the interlayer channels of graphene increases with the superimposition of the electrode size, reducing the lithium-ion storage performance of graphene electrodes. Additionally, it is difficult to ensure uniform dispersion of Si nanoparticles on the surface of graphene during the preparation process, and the exposed silicon nanoparticles are still easy to aggregate and decompose. Because of the difference in the volume expansion rate between silicon and graphene, the Si nanoparticles are extremely likely to be peeled off from graphene after several charge/discharge cycles, thereby resulting in a decrease in the capacity of LIB and a rapid decline in the electrode life characteristics in several cycles^26–28^. Therefore, to solve this problem, the manufacturing of a composite material with a stable structure is the main research direction at present.

In the case of a conventional electrode-manufacturing process, it is necessary to bond an electrode active material and a conductive agent to a metal current collector (Cu foil) with a polymer binder. However, because of the volume change of silicon, the material is crushed during the cycle and thus loses contact with the conductive agent and current collector. Consequently, it becomes difficult to form a stable SEI on the surface of the material, resulting in rapid dissipation of capacity. Additionally, for electrode materials with a high expansion rate, it is difficult to achieve the desired bonding effect with a general binder. If the amount of binder is increased, the internal resistance of the electrode will increase, resulting in a decrease in the energy density of the electrode^29–31^.

Therefore, in this study, a binder-free silicon nanoparticle/carbon nanofiber/graphene composite film was fabricated as an self-supporting negative electrode material. Carbon nanofibers have high specific surface area, high tensile strength and modulus of elasticity, excellent physical and chemical stabilities, excellent electrical conductivity and cycle characteristics, and high mechanical stability and durability over a wide range of temperatures and pressures. Carbon nanofibers are scattered around the silicon particles, and they can effectively accommodate and buffer the volume change of silicon and prevent the electrode structure from being damaged. Carbon nanofibers prevent Si particles from peeling from the carbon substrate upon the expansion of the surface area of Si particles, and they can prevent the direct contact between the Si nanoparticles and electrolyte; consequently, the Si particles can maintain their original volume size and form a stable SEI film. Furthermore, the addition of carbon nanofibers allows the composite film to form a fairly stable three-dimensional (3D) structure, which can effectively increase the specific surface area and provide an open channel for the immersion of the electrolyte. Additionally, the interlayer deposition of graphene is reduced; the transmission distance between electrons and lithium ions is shortened; the conductivity of the electrode material is improved; and the electrode possesses stable mechanical properties, excellent rate performance, and excellent cycle stability.

## Methods

### Materials

Iron (III) nitrate nonahydrates (Fe(NO_3_)_3_·9H_2_O, 98%), copper (II) nitrate trihydrates (Cu(NO_3_)_2_·3H_2_O, 99%), aluminum nitrate nonahydrates (Al(NO_3_)_3_·9H_2_O), ammonium molybdate tetrahydrates ((NH_4_)_6_Mo_7_O_24_·4H_2_O), and ammonium carbonate ((NH_4_)_2_CO_3_) were purchased from DAEJUNG CHEMICALS&METALS CO, Korea. All the reagents were of analytical grade and used as received. Silicon nanoparticles (powder, APS ≤ 50 nm, 98%) were purchased from Alfa Aesar, Inc. Graphene oxide (GO) was purchased from Angstron materials (Dayton, OH, USA N002-PS, 5.0%) and used as received. Ethyl alcohol (anhydrous, 99.9%) was purchased from Sigma-Aldrich. Deionized (DI) water (resistivity > 18 μΩ) was employed for preparing all the aqueous solutions for all the experiments.

### Synthesis of CNFs

We used co-precipitation to prepare Fe–Cu (70:30 at.%) bimetallic catalysts for the synthesis of carbon nanofibers. The preparation method of the catalysts is shown in Supplementary Fig. S1. First, we thoroughly mixed the aqueous solutions of A (Fe(NO_3_)_3_·9H_2_O + Cu(NO_3_)_2_·3H_2_O + Al(NO_3_)_3_·9H_2_O) and B ((NH_4_)_6_Mo_7_O_24_·4H_2_O). Subsequently, we added the mixture and C ((NH4)_2_CO_3_) aqueous solution dropwise into distilled water together and kept stirring to form a precipitate at room temperature at a pH of approximately 9.0. The precipitate solution obtained was vacuum filtered and oven-dried at 60 °C for 24 h. The dried precipitate was then crushed and ground into powder to be used as a metal catalyst for the synthesis of carbon nanofibers.

The Fe–Cu catalysts were used to synthesize carbon nanofibers by chemical vapor deposition (CVD). The synthesis process is shown in Supplementary Fig. S1. The catalyst powder was placed in a quartz reactor in a tube furnace, and the temperature was increased to 700 °C at a rate of 10 °C/min while maintaining an Ar gas flow. After reaching the target synthesis temperature, the Ar gas was replaced by a H_2_/Ar gas mixture during CVD to reduce the catalyst for 30 min. We then passed acetylene (C_2_H_2_), together with 10% H_2_/Ar gas mixture, as a carbon source into the reactor for 1 h. Finally, the reduced bimetallic catalysts were slowly cooled to room temperature while eliminating the supply of other gases but maintaining the Ar gas flow. In this way, carbon nanofibers can be successfully synthesized^32,33^.

### Preparation of Si/CNF/rGO and Si/rGO composites

**Figure 1:**
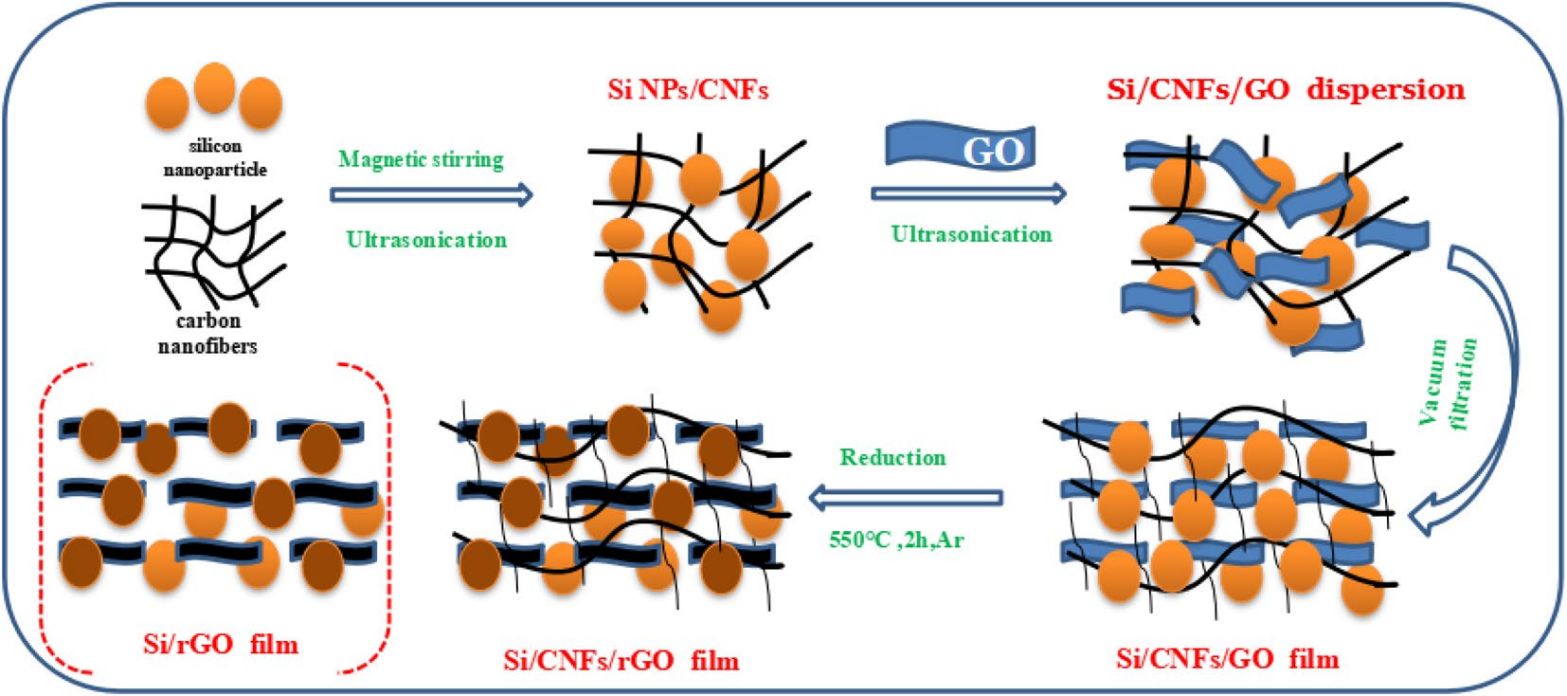
Preparation process of Si/CNF/rGO and Si/rGO composite films.

The synthesis process of Si nanoparticle/carbon nanofiber/graphene (Si/CNF/rGO) composite film is shown in Fig. 1. The prepared nano-silicon powder and CNFs were mixed and dispersed in ethanol under magnetic stirring for 1 h, and the mixture was then ultrasonically disrupted for 2 h to obtain a Si/CNF mixture. Subsequently, a GO solution was added to the mixture, and the resulting mixture was sonicated again for two hand magnetically stirred for 3 h to obtain a highly stable Si/CNF/GO dispersion. Afterward, the Si/CNF/GO dispersion was prepared by vacuum filtration to form a composite film with a total thickness of approximately 0.22 μm, and the film was dried in a blast oven at 60 °C for 24 h and then peeled off from the filter membrane to obtain a Si/CNF/GO composite film, which was then heated up to 550℃ at a rate of 10 °C/min in a quartz tube furnace with an argon flow maintained for 2 h to obtain thermally reduced Si/CNF/rGO composite film as the anode materials of LIB batteries. To optimize the electrochemical performances of the resultant materials, the composites were prepared by varying the mass ratio of the components, as Si:CNF/rGO = 3:2, 1:1, and 2:3. To compare the electrochemical performances of the resulting composites with one another, a control sample was prepared by using an Si/rGO film prepared by the same method without CNFs.

### Material characterization

The surface morphologies and micro-structures of the Si/CNF/rGO and Si/rGO composite films were characterized by field-emission scanning electron microscopy (FESEM, Hitachi S-4800) and transmission electron microscopy (TEM, JEM-2100). The qualitative and quantitative analyses of the prepared samples were performed by energy dispersive X-ray spectroscopy (EDS, Thermo ARL, ARL-3460). The characterizations of the sample composition and crystal structure were performed by taking power X-ray diffraction (XRD) measurements using an Ultima IV, 2 kW system with Cu-Kα radiation (K = 1.5418 Å), scanned with the 2θ range from 2° to 90°. Raman spectroscopy analysis were performed on a Horiba Jobin–Yvon (Raman, LABRAM HR-800) with a laser light (λ = 514 nm) in a wave number range of 100–3000/cm. The amounts of SiNPs, rGO, and CNFs in the composites were measured by thermo-gravimetric analysis (TGA) using the Perkin Elmer Diamond TG/DAT thermal analyzer, from room temperature to 800 °C at a heating rate of 10 °C/min under an air atmosphere. X-ray photoelectron spectroscopy (XPS, Thermo Fisher Scientific, Multilab-2000) analysis was conducted on a twin anode with Al Kα radiation as an X-ray source.

### Fabrication of LIBs and electrochemical measurements

In this study, two-electrode batteries were prepared using Si/CNF/rGO and Si/rGO composite materials as negative electrode active materials for LIBs. To test the electrodes and characterize their electrochemical performances, the prepared Si/CNF/rGO and Si/rGO composite films are cut into small pieces and used as independent working electrode materials without using conductive additive and binders. Metallic lithium foil as the counter and reference electrodes, and two-electrode lithium-ion coin cells (CR2032) were assembled in a high-purity argon-filled glove box. The separator membrane was Celgard 2600, and the electrolyte was a solution of 1 M LiPF_6_ dissolved in a mixture of ethylene carbonate (EC)/dimethyl carbonate (DMC)/ethyl methyl carbonate (EMC) (1:1:1 by volume). Cyclic voltammetry (CV) and galvanostatic charge–discharge measurements were taken at room temperature (25 °C) by using an electrochemical work-station and a battery tester (Neware Co., Ltd. SHENZHEN, CHINA) at the scan rate of 0.1 mV/s between the voltage range of 0.01–1.5 V (vs.Li/Li+). The number of cycles of the charge/discharge experiment was measured as 100. Electrochemical impedance spectroscopy (EIS) measurements were taken on a CHI 660D electrochemical analysis instrument (CH Instruments, Inc. SHANGHAI, CHINA) between the frequency ranging from 100 kHz to 10 MHz with an amplitude of 5 mV. For comparison, a control-group Si/rGO composite electrode was prepared and tested under the same conditions.

## Results and discussion

### Structure and morphology

**Figure 2:**
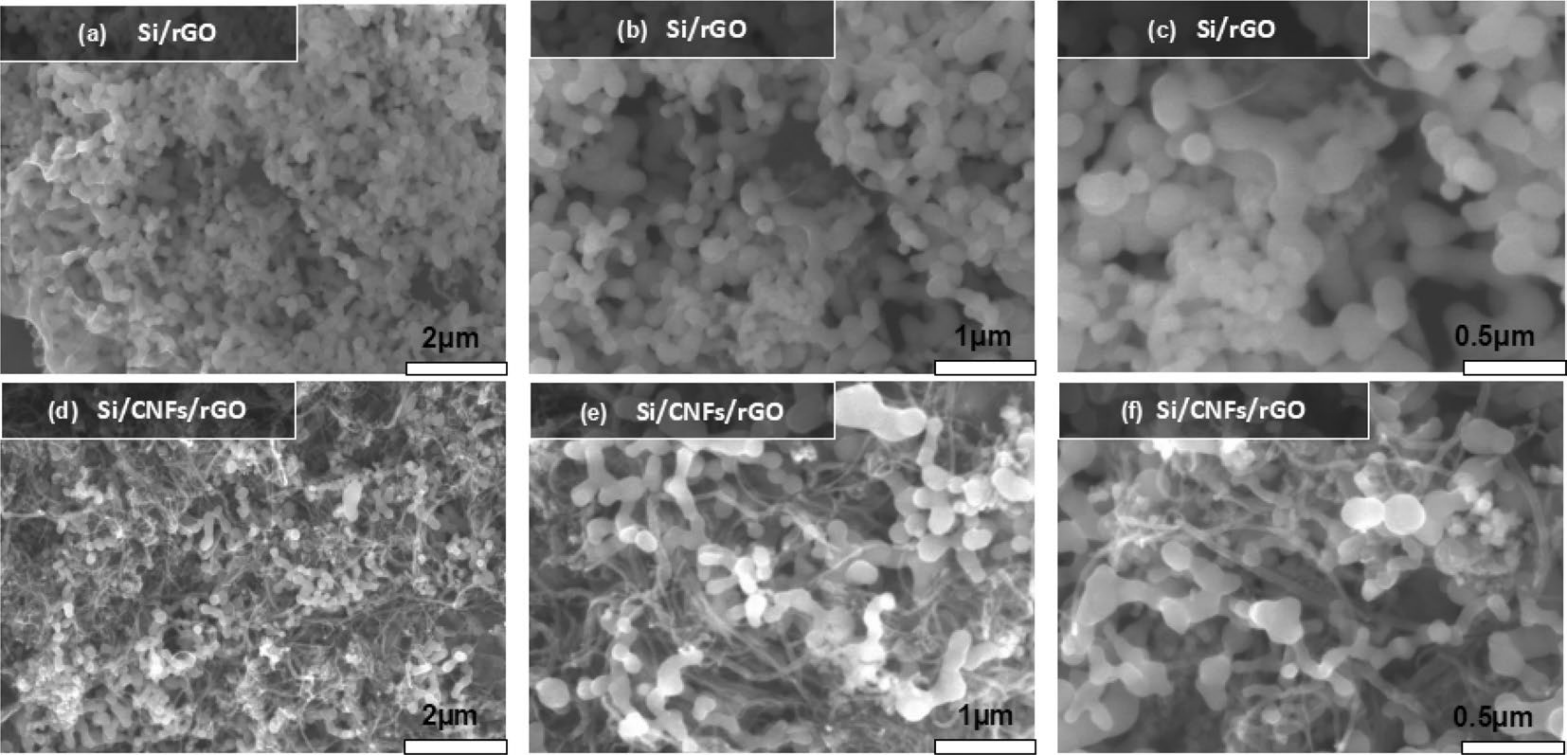
SEM images of the (**a**–**c**) Si/rGO and (**d**–**f**) Si:CNF/rGO = 1:1 composites.

Figure 2 shows the SEM images of the Si:CNF/rGO = 1:1 composite film and Si/rGO composite film. The EDX spectral results of Si:CNF/rGO = 1:1, Si:CNF/rGO = 3:2, and Si:CNF/rGO = 2:3 are presented in Supplementary Table S1. It can be clearly seen from Fig. 2a,b that the silicon nanoparticles covered with the graphene coating are uniformly dispersed in the carbon nanofiber grid. From Fig. 2c, it can be seen that the Si/rGO composite is densely entangled by carbon nanofibers, and that the carbon nanofibers are interspersed among the silicon particles to form a stable three-dimensional structure. Notably, this structure provides a short transport channel for the electron and ion transport, thereby imparting a good electrical conductivity and low electrical resistance to the entire electrode^34^. Additionally, the three-dimensional grid structure has good number of voids, which can effectively accommodate the volume change of the entire electrode structure and reduce the fragmentation of Si nanoparticles during the charging and discharging processes, thereby providing an excellent mechanical integrity to the electrode. The SEM images of the Si:CNF/rGO = 1:1 composite film as a control electrode are shown in Fig. 2d–f. The Si nanoparticles are covered with a graphene layer, and the close contact between the Si nanoparticles and reduced graphene coating means that a stable composite structure is formed.

**Figure 3:**
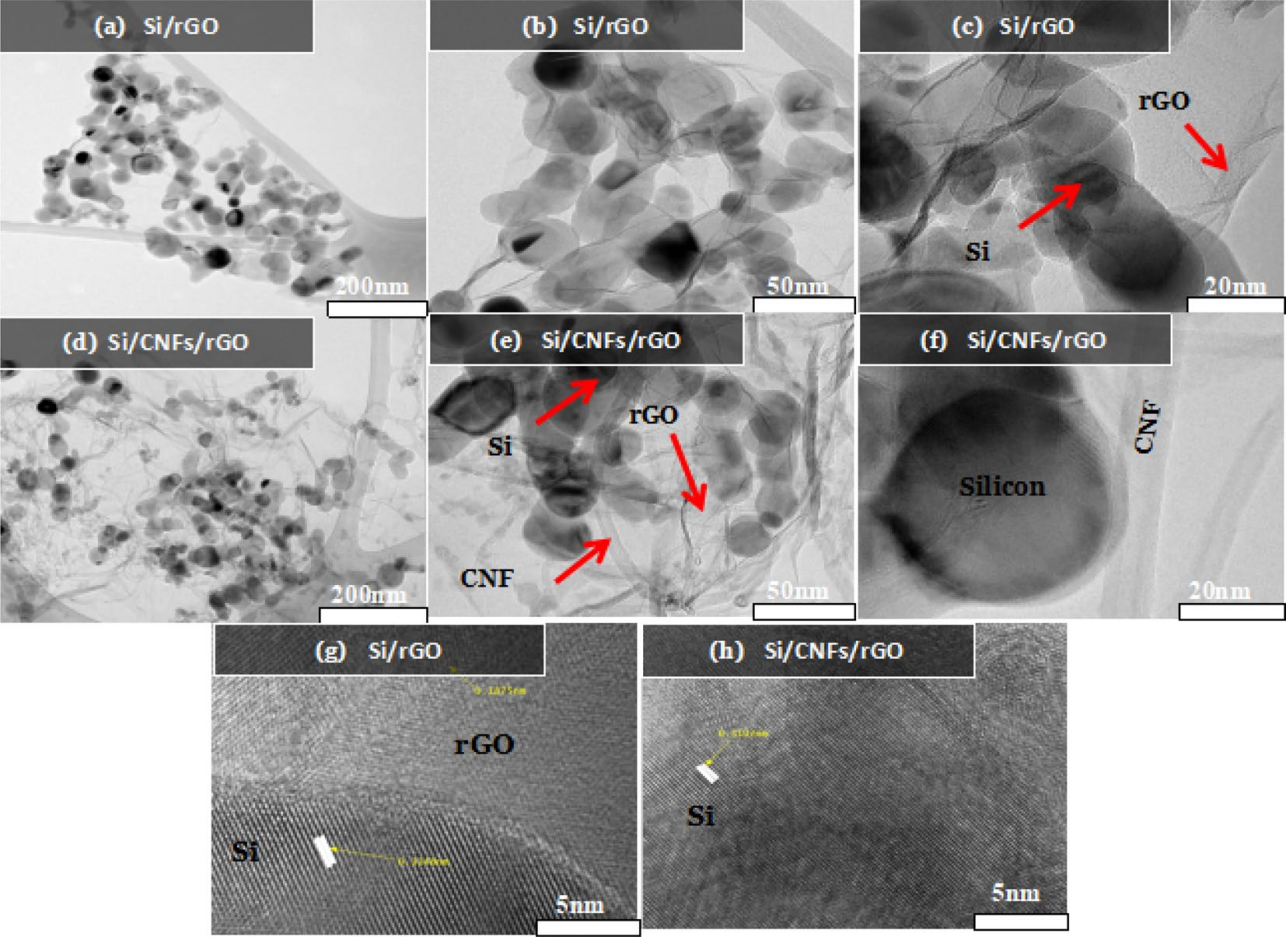
TEM images of (**a**–**c**) Si/rGO, (**d**–**f**) Si:CNF/rGO = 1:1 composites. High-resolution TEM images of (**g**) Si/rGO and (**h**) Si:CNF/rGO = 1:1 composites.

To further investigate the structures and compositions of the Si:CNF/rGO = 1:1 and Si/rGO composite films, TEM analyses were performed, as shown in Fig. 3. The TEM images of the Si/rGO composite film is shown in Fig. 3a–c. As can be seen from Fig. 3a,b, Si/rGO particles were distributed in a cross-linked carbon nanofiber grid, and graphene and carbon nanofibers formed a woven structure with each other. As can be seen from Fig. 3c, the surface of the Si nanoparticles is uniformly covered with graphene coating. The coating of the carbon layer and the entanglement of CNFs with the Si nanoparticles indicate that the volume change of the silicon particles can be effectively accommodated and buffered, thereby preventing the surface cracking of the silicon particles due to their volume change and also preventing the peeling off of the silicon particles from the carbon substrate, eventually facilitating the formation of a stable SEI film to maintain the integrity of the electrode structure. As an anode material, the stable structure will contribute to achieving a high specific capacity and excellent cycle and rate performances. Figure 3d–f shows the TEM images of the Si:CNF/rGO = 1:1 composite film, and the corrugated graphene sheet and Si nanoparticles can be clearly seen. Si nanoparticles and rGO flakes are evenly distributed throughout the film, and the graphene sheets are tightly coated onto the Si nanoparticles to accommodate the volume change of the Si particles. The result is consistent with the SEM images. The result is beneficial to improving the electrical conductivity of the composite material. In Fig. 3g,h, the high-magnification TEM images show that the lattice spacing of Si NP is 0.31 nm, which corresponds to Si (111) surface and the lattice spacing of rGO is 0.18 nm.

**Figure 4:**
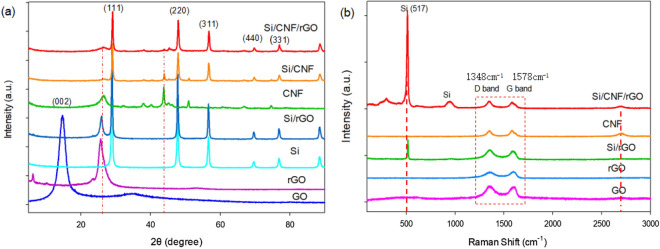
(**a**) X-ray diffraction patterns of Si/CNF/rGO, Si/CNFs, CNFs, Si/rGO, Si, rGO, and GO. (**b**) Raman spectra of the Si/CNF/rGO composite, CNFs, Si/rGO composite, rGO, and GO.

The XRD patterns of Si/CNF/rGO, Si/CNF, Si/rGO, CNFs, rGO, and GO are shown in Fig. 4a. In the case of GO, a strong and narrow representative diffraction peak appeared at 2θ = 9.6°. In the curve of thermally reduced graphene, the disappearance of the 9.6° characteristic peak of GO and after the reaction and the regeneration of the wide and weak 2θ = 25° (002) peak corresponding to graphene was regenerated, indicating that GO had been successfully converted to rGO^35–37^. The diffraction peaks of the Si/rGO film appeared at 26.5° and 28.4°, which confirmed the existence of rGO and Si nanoparticles, respectively. Additionally, the characteristic peaks of crystalline silicon appeared at Si (111) 28.4°, Si (220) 47.3°, Si (311) 56.1°, Si (400) 69.1°, and Si (331) 76.4° (JCPDS 27-1402). Peaks also appeared in Si/CNF/rGO, meaning that there was no change in Si even after combination with rGO and CNFs. Additionally, the characteristic peaks of CNFs at 26.78°, 43.84°, and 66.87° were also shown in Si/CNF/rGO. A weak peak appeared near 24°, which corresponds to the characteristic peak position of amorphous carbon.

Figure 4b shows the Raman spectra of the Si/CNF/rGO composite. Additionally, the spectral analyses of GO, rGO, CNFs, and rGO–Si composites were performed, and the results are presented in Supplementary Table S2. From the curve results of the Si/CNF/rGO and Si/rGO composite films, it is evident that the peak at approximately 517/cm coincided with the data of the Si nanoparticles spectrum^39^, indicating that the volume of the Si nanoparticles undergoes no change after bonding to the composite. The D and G bands are located at 1348 and 1578/cm, respectively, which are the characteristic peaks of disordered carbon and sp^2^ hybrid carbon materials, respectively. The ID/IG intensity ratio is a parameter for evaluating the degree of graphitization of carbon materials and the density of defects in graphene-based materials. Compared with GO (1.09), the ID/IG intensity ratios of Si/CNF/rGO (1.02), Si/rGO (1.04), and rGO (0.96) decreased after thermal reduction. This is because the size of the in-plane sp^2^ region in the plane significantly increased, and the intensity of the D-band decreased as the degree of carbonization increased^38,39^. Additionally, because CNFs (0.99) have a few defects, adding CNFs will weaken the D-band, which is conducive to enhancing the conductivity of the composite material.

**Figure 5:**
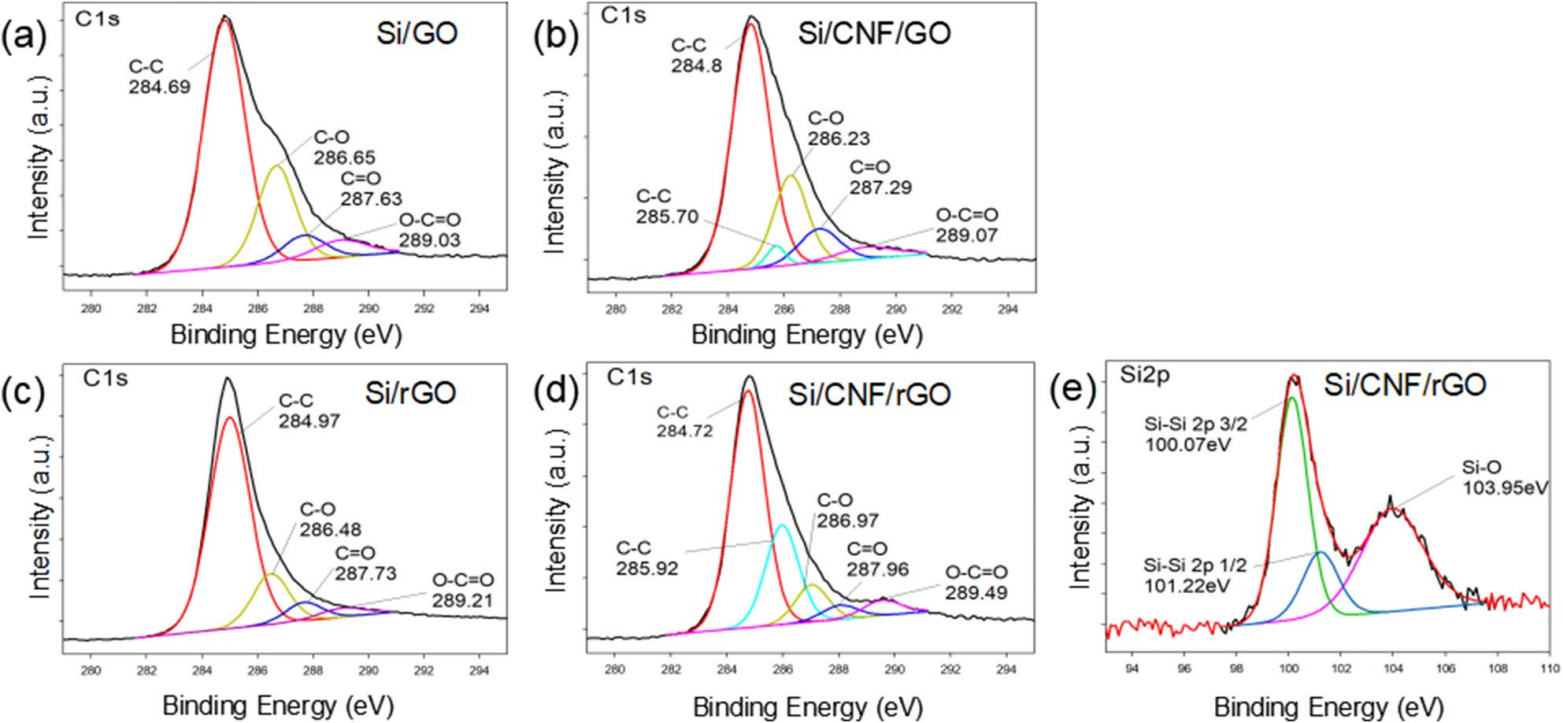
C 1 s XPS spectra of (**a**) Si/GO, (**b**) Si/CNFs/GO, (**c**) Si/rGO, and (**d**) Si/CNF/rGO. (**e**) Si 2p XPS spectrum of Si/CNF/rGO.

The elemental information of the Si/CNF/rGO composite material was analyzed by XPS, as shown in Fig. 5. In the C1s XPS spectra of the Si/GO, Si/CNFs/GO, Si/rGO, and Si/CNF/rGO composite materials (see Fig. 5a–d), the strong peaks at approximately 284 and 286 eV represent the existence of C–C and C–O bonds, respectively. The weak peaks at 287 and 289 eV correspond to the existence of C=O and O–C=O bonds, respectively. However, because of the low contents of O–C=O and C=O bonds, the peak intensity is also weak^40,41^. By comparing Fig. 5a,c, it can be seen that the C–O bond in the spectrum of composite Si/GO changes from a strong peak to a weak peak in the spectrum of composite Si/rGO, which is caused by the loss of oxygen after thermal reduction. The results of Fig. 5b,d also show the same trend. Comparing Fig. 5c with Fig. 5d, it can be seen that because of the addition of CNFs, the spectrum in Fig. 5d has a peak at 285.9 eV, which corresponds to the C–C bond in CNFs. Additionally, it can be seen that because of the thermal reduction of GO to rGO, the intensity of the C–C bond peak between rGO and CNFs at 285 eV has decreased. In Fig. 5e, three Si 2p peaks appear. The peaks at 100.07 and 101.22 eV correspond to Si–Si 2p3/2 and Si–Si 2p1/2, respectively, while that at 103.95 eV corresponds to the Si–O bond. The Si–O bond appeared because a small amount of nano-scale silicon powder was exposed to air and oxidized at a certain temperature during the manufacturing process of the material.

**Figure 6:**
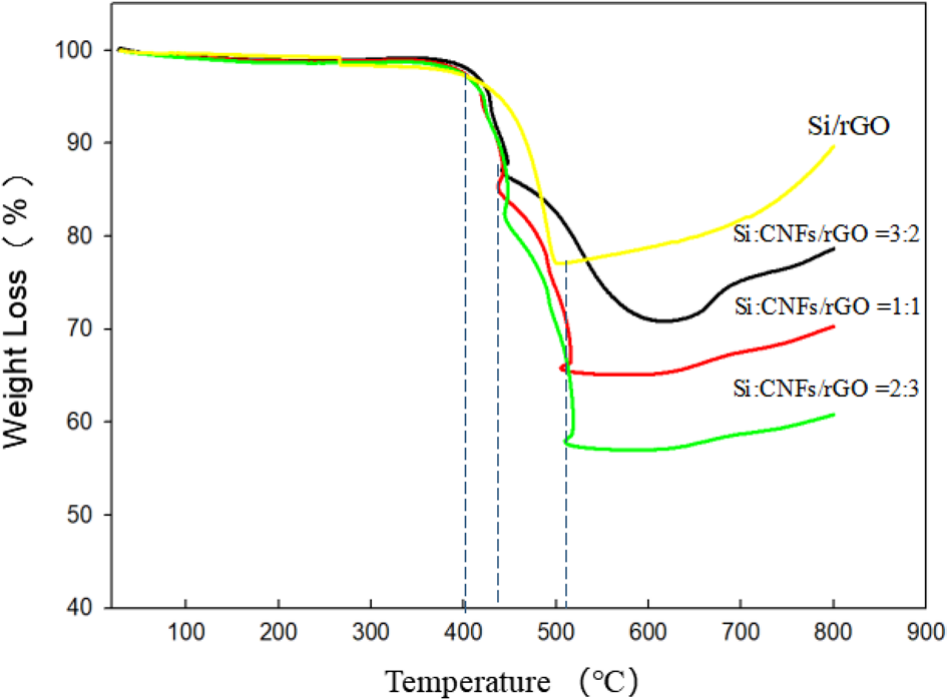
TGA curves of the Si/rGO, Si:CNF/rGO = 3:2, Si:CNF/rGO = 1:1, and Si:CNF/rGO = 2:3 samples.

Figure 6 shows the TGA curve of each Si/CNF/rGO composite with different component contents. The weight of the Si/CNF/rGO composite material rapidly decreased from 420 to 520 °C, indicating that rGO and CNFs were rapidly oxidized and decomposed in air. Between rGO and CNFs, it was confirmed that graphene were first decomposed between 420 and 440 °C. The decrease in the sample weight between 440 and 520 °C is attributed to the decomposition of CNFs. Through the decrease of the sample curve, it can be confirmed that the Si content in each composite film of Si:CNF/rGO = 3:2, 1:1, and 2:3 can reach 71.0 wt%, 64.25 wt%, and 56.9 wt%, respectively. The total content of rGO and CNF can be measured by the reduction in total weight.

We separately analyzed the TGA curve of the Si:CNF/rGO = 1:1 sample in detail, as shown in Supplementary Fig. S2. According to the results of the TGA test in air, the weight gradually increased after 520 °C, although the upward trend of the TGA curve was unclear. This is because a small amount of SiO_2_ was formed on the surface of Si nanoparticles because of the oxidation reaction^42^. This result is consistent with the result of the Si 2p spectrum of XPS.

### Electrochemical performance

**Figure 7:**
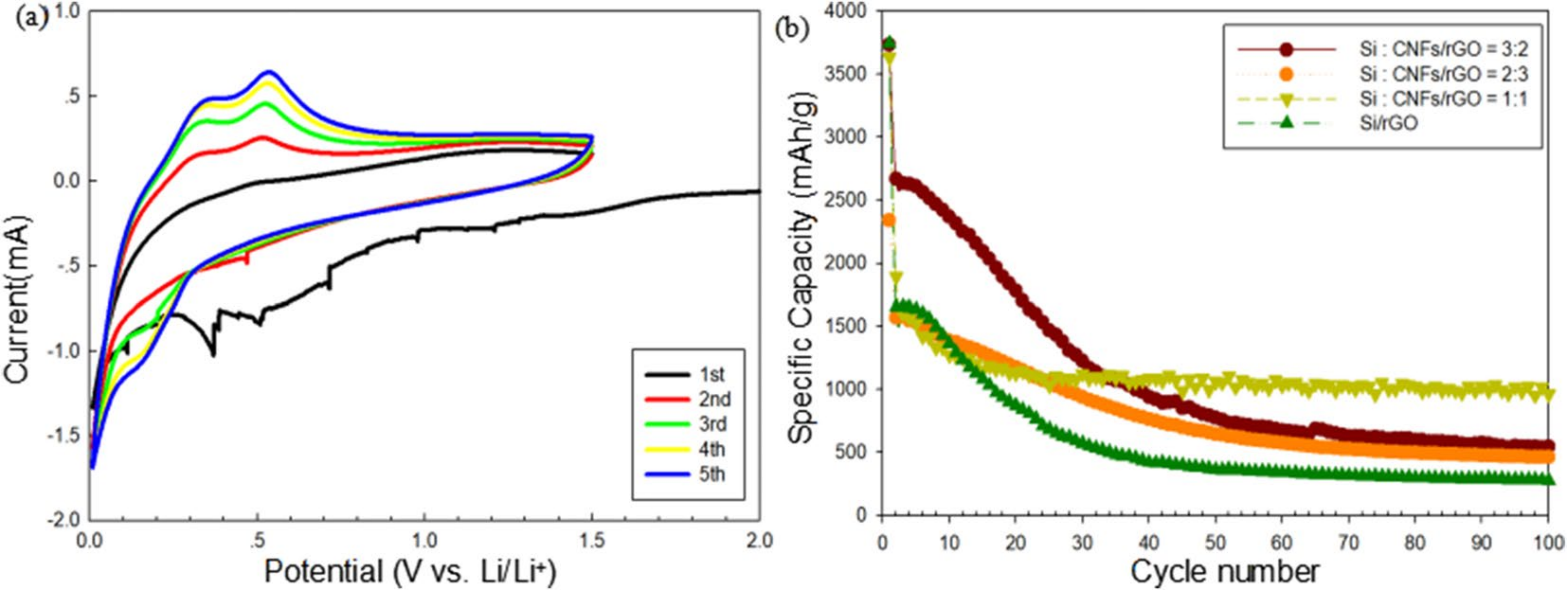
(**a**) Cyclic voltammetry curves of the synthesized Si/CNF/rGO = 1:1 composite in the initial five cycles. (**b**) Cycling performances of the synthesized Si/rGO and Si/CNF/rGO = 1:1, 2:3, and 3:2 composite electrodes at a current density of 0.1 A/g.

In the voltage window of 0.01–1.5 V (vs Li^+^/Li), the cyclic volt-ampere curve (CV) of a coin cell fabricated by the Si:CNF/rGO = 1:1 electrode was tested in the first five cycles at a scan rate of 0.1 mV/s, as shown in Fig. 7a. It can be seen that in the CV curve of the first cycle, a broad and weak cathode peak of 0.98 V appears in the cathode scan; the peak may be related to the reaction between the electrode material and electrolyte and the formation of an irreversible SEI film on the electrode surface. However, this cathode peak disappeared in the subsequent cycles, indicating that after the first cycle, a stable SEI film was formed on the electrode material surface. The strong reduction peak at around 0.25 V corresponds to the amorphous Li_x_Si alloy formed by amorphous silicon during reversible lithium-ion insertion/extraction. During the delithiation process, the two oxidation peaks at 0.35 and 0.54 V correspond to the decomposition of the Li_x_Si alloy into amorphous silicon^43^. Additionally, as the number of scans increases, the intensity of the anode peak also gradually increases, indicating that the composite electrode material was gradually activated during the cycle. This is consistent with previous reports^44,45^.

Figure 7b shows compares the cycle performances of the four samples prepared at a current density of 0.1 A/g. The charge and discharge capacity values, coulombic efficiency, and capacity retention rate of each sample are presented in Supplementary Table S3. Although both Si/rGO and Si/CNF/rGO composites have high initial capacity (approximately 3743.8 mAh/g), the extremely high capacity loss in the first cycle may be attributed to the irreversible processes, such as electrolyte decomposition and unavoidable SEI layer formation. It can be seen that the specific capacity of the Si/rGO electrode material (see Supplementary Fig. S3a) rapidly decreases to 277.3 mAh/g after 100 cycles. This is because the graphene-coated composite electrode still cannot adapt to the enormous change in the volume of the Si particles during the lithiation/delithiation process, thereby resulting in the formation of a thick SEI layer on the electrode surface; this thick SEI layer interferes with the conduction of electrons and eventually leads to a gradual decrease in the battery capacity. However, compared with the Si/rGO composite electrode, the Si/CNF/rGO composite electrode shows a better cycle performance. Among the three Si/CNF/rGO composite electrodes with different composition contents, the Si:CNF/rGO = 3:2 electrode, owing to its highest content of silicon nanoparticles, possesses the highest initial capacity of 3734.3 mAh/g; however, as the number of cycles increases, the capacity significantly decays to only 545.9 mAh/g after 100 cycles. The charge and discharge curve (see Supplementary Fig. S3b) shows that although the Si:CNF/rGO = 3:2 electrode has a fairly high initial capacity, its first discharge capacity is very low, and also the coulomb efficiency is fairly low. However, the coulombic efficiency can reach 99.9% after several cycles because the contents of CNFs and graphene in the electrode are low. Moreover, the conductivity of the Si:CNF/rGO = 3:2 electrode is poor, and a large number of silicon nanoparticles are easy to aggregate, resulting in a poor cycle stability. For the Si:CNF/rGO = 2:3 composite electrode (see Supplementary Fig. S3c), the initial capacity (2339.1 mAh/g) and reversible specific capacity (462.9 mAh/g) after 100 cycles are the lowest, although its cycle performance is the best. Because its CNFs and graphene content are high, it has the most stable electrical conductivity. Additionally, its coulombic efficiency can also reach 100%. With increase in the graphene content, the specific capacity of the electrode is reduced, although the cycle stability is significantly improved. This is consistent with previous reports. The content of silicon in the composite material significantly affects the conductivity and actual capacity of the composite electrode^46^. However, the Si:CNF/rGO = 1:1 composite electrode is the best electrode, as it has the highest specific capacity and best cycle stability. Even after 100 cycles, the Si:CNF/rGO = 1:1 composite electrode maintains a high specific capacity of approximately 964.68 mAh/g. The initial coulombic efficiency of the Si:CNF/rGO = 1:1 composite electrode is only 26.0%, which is fairly low because the contact between the electrolyte and graphene forms the SEI layer. However, after three cycles, the coulombic efficiency increased to 93.8% (see Supplementary Fig. S3d). Obviously, the excellent cycle performance of the Si:CNF/rGO = 1:1 composite electrode is attributed to the addition of a graphene coating, which improves the conductivity of the electrode and prevents direct contact between the electrolyte and silicon particles, thereby facilitating the formation of a stable SEI layer. The added CNFs can prevent the accumulation of a large amount of silicon nanoparticles and GO during the manufacturing process, thereby effectively increasing the contact area between rGO and electrolyte. CNFs act as three-dimensional cross-linked structures between graphene and silicon, thereby preventing the desorption of the graphene layer generated during the volume expansion and contraction of the Si nanoparticles, and improving conductivity, eventually resulting in high capacity and cycle stability. Additionally, the three-dimensional structure provides a quick and effective channel for electron and ion transport. It can be seen from the results that as the total contents of CNFs and reduced GO added to the silicon nanoparticles increases, the electrode capacity of the composite electrode after 100 cycles also decreases. Due to the difference in silicon content in the composite electrode, the initial capacity of the electrode is also different.

**Figure 8:**
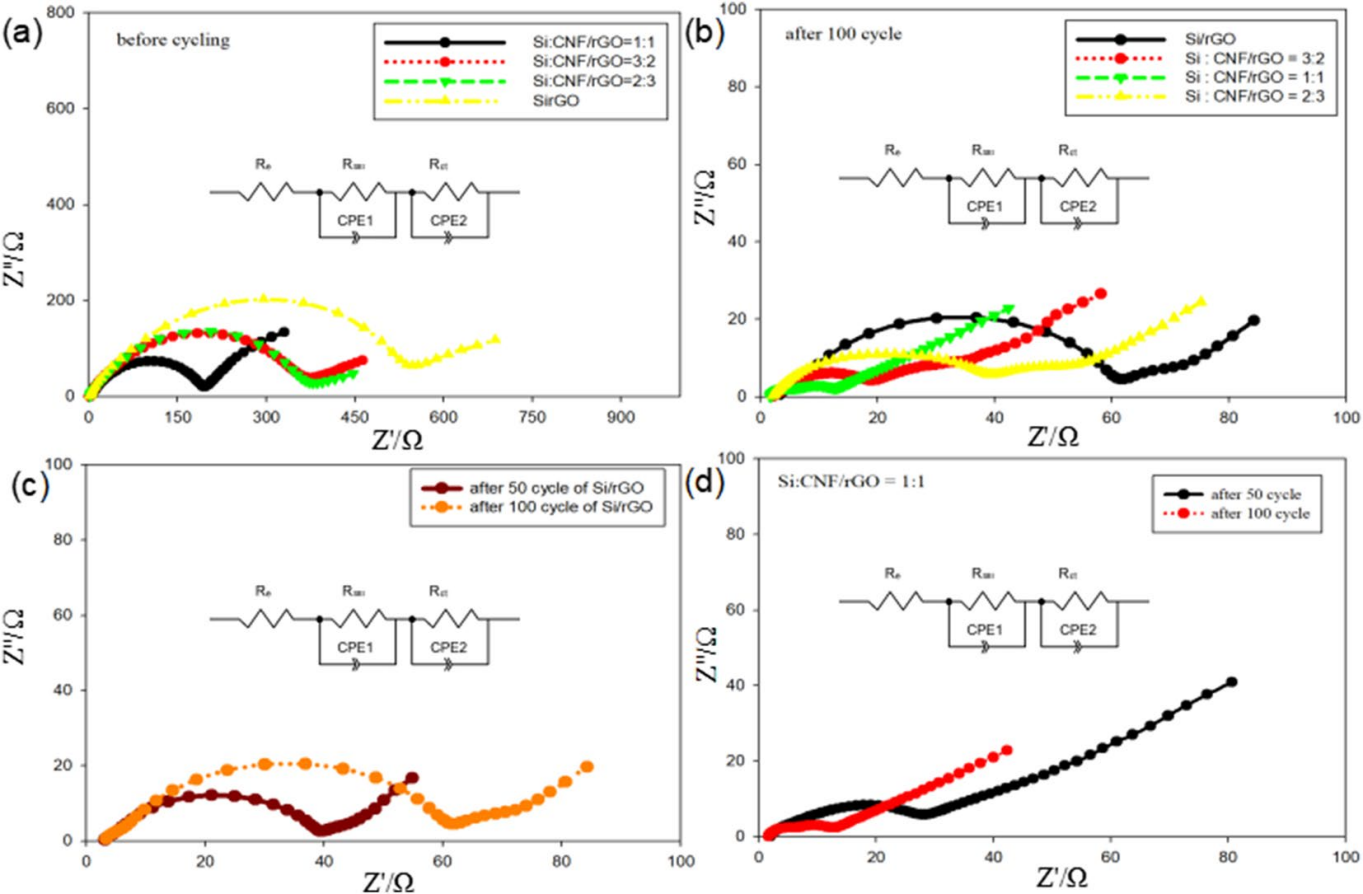
(**a**) Nyquist plots and electrochemical impedance spectra of the Si:CNF/rGO = 1:1, 3:2, and 2:3 and Si/rGO composite anodes before cycling. (**b**) Nyquist plots and electrochemical impedance spectra of the Si:CNF/rGO = 1:1, 3:2, and 2:3, and Si/rGO composites anode after cycling. (**c**, **d**) EIS curves of the Si/rGO composites anode (**c**) and Si:CNF/rGO = 1:1 composite anode (**d**) after the 50th and 100th cycles.

To better understand the chemical reaction kinetics of each sample, the EIS patterns of different electrodes at frequencies ranging from 10 MHz to 100 kHz and amplitude ratios of 5 mV were investigated, as shown in Fig. 8. We can see that all the curves in the Nyquist plots appear as a semicircle in the high-frequency and middle-frequency regions, and appear as a slanted line in the low-frequency region. The diameter of the semicircle is related to the resistance of lithium ions through the insulating layer on the surface of the active material particles (R_SEI_) and the charge-transfer resistance (R_CT_). The slanted line corresponds to the diffusion resistance of lithium ions within the electrode active material. The diffusion resistance is correspondingly expressed as Warburg impedance (Z_W_)^47^. Figure 8a shows the Nyquist plots of the Si/rGO and Si/CNF/rGO electrodes after cycling. Expectedly, the R_CT_ values of the Si:CNF/rGO = 1:1, 2:3, and 3:2 composites are only 247.3, 372.0, and 374.3 Ω, respectively, and the R_CT_ value of Si/rGO is 546.8 Ω. The charge-transfer resistance of the Si:CNF/rGO = 1:1 composite electrode is significantly lower than those of the other composite electrodes, showing that the Si:CNF/rGO = 1:1 electrode layer can effectively enhance the transfer of electrons and charges, thereby significantly reducing the charge-transfer resistance and improving the electrochemical performance of the electrode.

To further evaluate whether a stable SEI film was formed, the Nyquist plots of all the samples after 100 cycles were investigated, as shown in Fig. 8b. The Si:CNF/rGO = 1:1 electrode showed an R_SEI_ value of 12.0 Ω, which is lower than those of the Si:CNF/rGO = 3:2 (17.9 Ω), Si:CNF/rGO = 2:3 (36.7 Ω), and Si/rGO (58.2 Ω) composite electrodes. This indicates that the insertion of CNFs is beneficial to reducing the formation of the SEI film on the electrode surface, thereby reducing the resistance value. Figure 8d compares the Nyquist plots of the Si:CNF/rGO = 1:1 electrodes after 50 and 100 cycles, respectively, with the resistance R_SEI_ values of 26.3 and 12.0 Ω, respectively. To draw comparison among the excellent performances of the active electrodes analyzed in this study, we measured the R_SEI_ values of the Si/rGO electrode after 50 and 100 cycles respectively, which results in the resistance values of R_SEI_ = 36.8 Ω and R_SEI_ = 58.2 Ω, respectively. As shown in Fig. 8c, as the number of cycles increases, the resistance value does not significantly change, and the SEI layer tends to stabilize. This shows that the electrode structure has not significantly changed, and that the electrode has a good structural stability, thereby providing an effective channel for lithium-ion transfer, eventually improving the conductivity of the electrode. The R_CT_ and R_SEI_ values calculated from the EIS spectra are listed in Supplementary Table S4. A comparison between the Nyquist plots of the Si:CNF/rGO = 2:3 and Si:CNF/rGO = 3:2 electrodes after 50 and 100 cycles is shown in Supplementary Fig. S4. Therefore, we conclude that the resistance value of the composite electrode will depend on the content of silicon nanoparticles. The higher is the content of silicon nanoparticles, the higher is the R_CT_ value.

To better understand the cycle performance of the composite materials, this paper also compares the surface SEM images of the Si/CNF/rGO composite electrode material after 100 cycles and the electrode material before the cycle. Under low magnification, both the electrode surface before the cycle and the electrode after 100 cycles showed a rough texture (Fig. S5a,d). However, the original silicon particles, graphene, carbon nanofibers, and other structures cannot be clearly observed on the electrode surface after the cycle. This is because the products of some side reactions, which occur during the cycle accumulate on the electrode surface, covering active materials such as silicon particles^48^. Comparing Fig. S5b,e, no obvious cracks in the silicon-based anode material are observed in the image, and the silicon nanoparticles are uniformly distributed. This is significantly different from the previously reported SEM image results of silicon-based materials as anode materials for lithium-ion batteries^49^. These results indicate that the combination of graphene and carbon nanofibers creates a certain flexible space that can accommodate the volume change of silicon particles during repeated lithiation/delithiation processes, and inhibits the cracking of the active material, thereby preventing the electrode from breakage. The integrity of the electrode is protected to a certain extent. Figure S5c,f show a further enlarged view of the electrode surface topography. For the electrode structure after cycling, it can be clearly seen that the edge of the graphene sheet exhibits a roughly circular surface, which may be related to the formation of the SEI layer. The SEI layer may be due to the fact that the edge portion of the graphene is crushed during the volume change of the silicon particles or is related to the product of lithium electroplating and side reactions generated in the main reaction between the surface of the active material and the electrolyte^50^. This leads to poor contact between the active materials, and a the thickness of SEI film will also increase the resistance of the electrode, thereby affecting the cycle performance of the sample.

## Conclusions

In this study, we successfully synthesized CNFs and then prepared silicon nanoparticle Si/CNF/ rGO composite electrodes through simple physical mixing and thermal reduction. Through the characterization of the samples, it was confirmed that rGO was uniformly encapsulated on the surface of the silicon nanoparticles, and that the carbon nanofibers were wound around the Si/rGO to form a three-dimensional structure of the Si/CNF/rGO composite. When the Si/CNF/rGO composite film was used as an anode material for LIBs, it exhibited an excellent electrochemical performance, high cycle stability, and high reversible specific capacity. When the current density was 0.1 A/g, the following observations were made: even after 100 cycles, the initial specific capacity was 1894.54 mAh/g, specific capacity maintained at 964.68 mAh/g, and coulombic efficiency 98.7%. The three-dimensional structure of the Si/CNF/rGO composite, on the one hand, maintains the stability of the electrode; on the other hand, it provides a fast and effective channel for electron and ion transport. The graphene coated on the surface of the silicon nanoparticles not only improved the conductivity of the electrode, but also prevented direct contact between the electrolyte and silicon nanoparticles, thereby forming a stable SEI film. Additionally, the graphene coating can effectively adapt to the volume expansion and contraction of the silicon nanoparticles and prevent the silicon nanoparticles from being broken during charge and discharge cycles.

## Supplementary Information

Supplementary Information.

## Data Availability

The datasets generated during and/or analyzed during the current study are available from the corresponding author on reasonable request.
