# Supplementary material for: Characteristics and electrochemical performances of silicon/carbon nanofiber/graphene composite films as anode materials for binder-free lithium-ion batteries
Source: Sci Rep. 2021 Jan 14;11:1283. doi: 10.1038/s41598-020-79205-1 (PMC7809343; doi:10.1038/s41598-020-79205-1)
Supplement: Supplementary file 1 — Supplementary Information. [file 41598_2020_79205_MOESM1_ESM.docx]

**Supplementary Information**

Characteristics and Electrochemical Performances of Silicon/Carbon nanofiber/Graphene Composite Films as Anode Materials for Binder-Free Lithium-Ion Batteries

Ruye Cong^a^, Jin-Yeong Choi^a^, Ju-Beom Song^b^, Minsang Jo^C^, Hochun Lee^C^,

Chang-Seop Lee^a,*^

^a^Department of Chemistry, Keimyung University, Daegu 42601, Korea

^b^Department of Chemical Education, Kyungpook National University, Daegu 41566, Korea

^C^Department of Energy Science and Engineering, DGIST, Daegu 42988, Korea

^*^Corresponding author: Chang-Seop Lee

Department of Chemistry, Keimyung University, Daegu 42601, Korea

Phone No: +82-53-580-5192; Fax No: +82-53-580-5056

E-mail: [surfkm@kmu.ac.kr](mailto:surfkm@kmu.ac.kr)


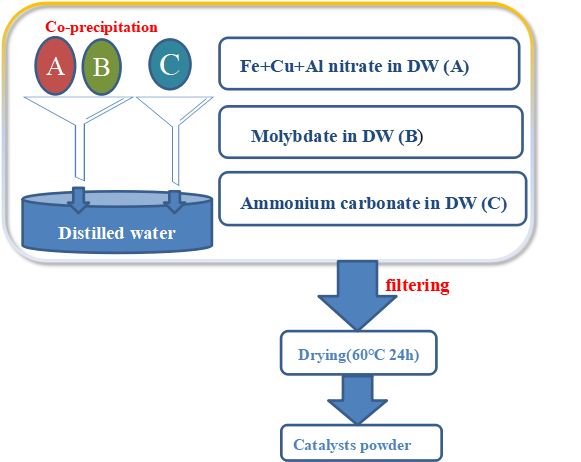

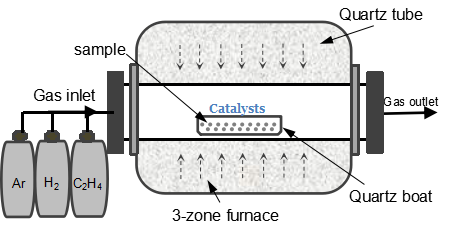

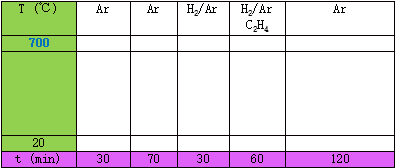


**Fig. S1.** Process for preparing the catalysts and the synthesis of carbon nanofibers.


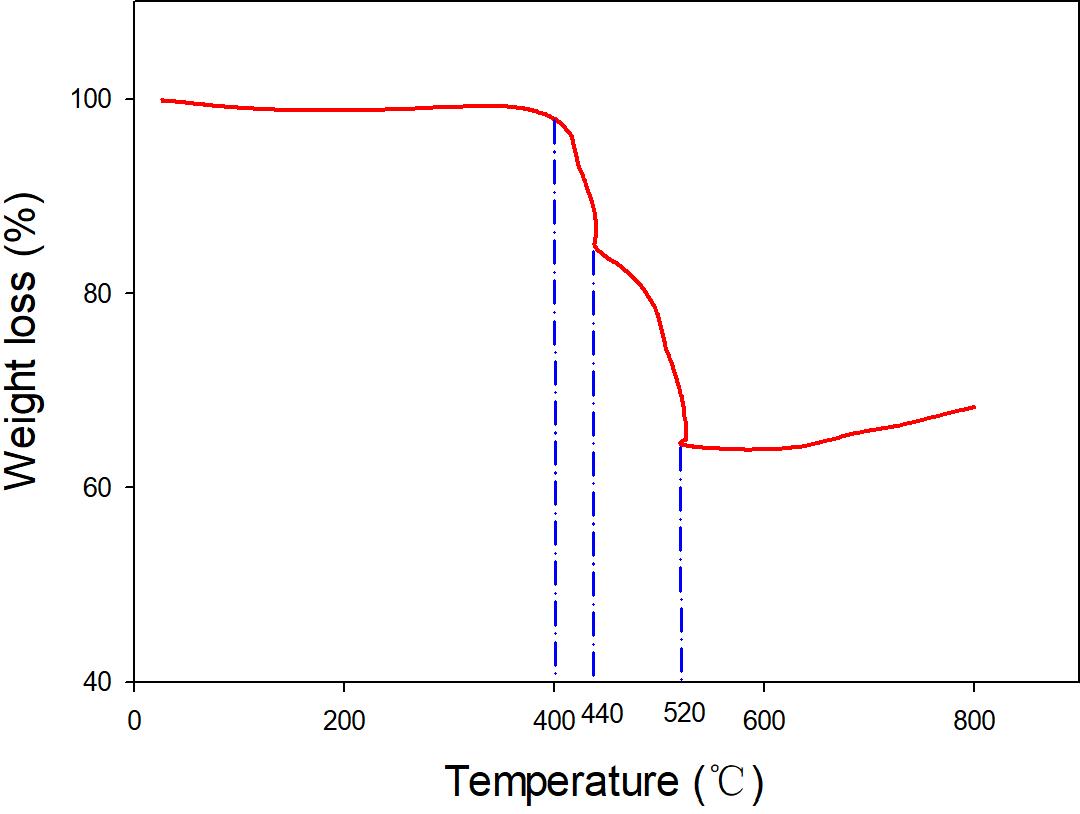


**Si:64.25 wt%**

**CNFs:20.75 wt%**

**rGO:15 wt%**

rGO

CNFs

**Fig. S2.** TGA curves of the Si:CNF/rGO =1:1 composite.


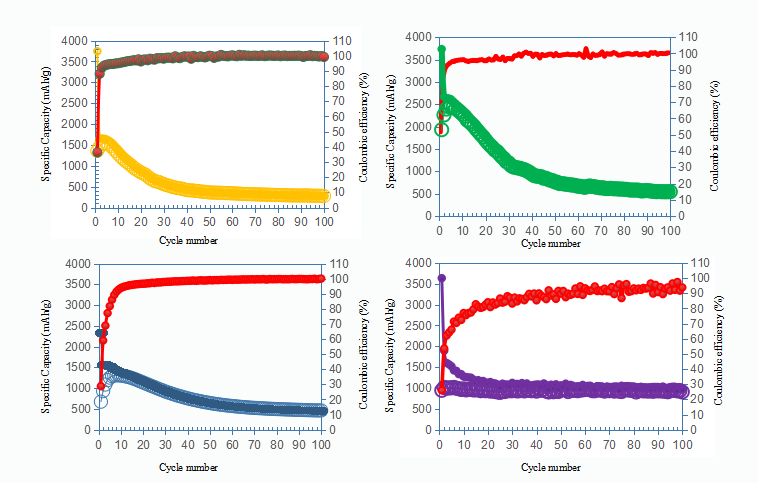


a

d

c

b

**Fig. S3.** Cycling performances of the synthesized Si/rGO (a), Si:CNF/rGO = 3:2 (b), Si:CNF/rGO = 2:3 (c), and Si:CNF/rGO = 1:1 (d) composite electrodes at a current density of 0.1A·g^−1^.


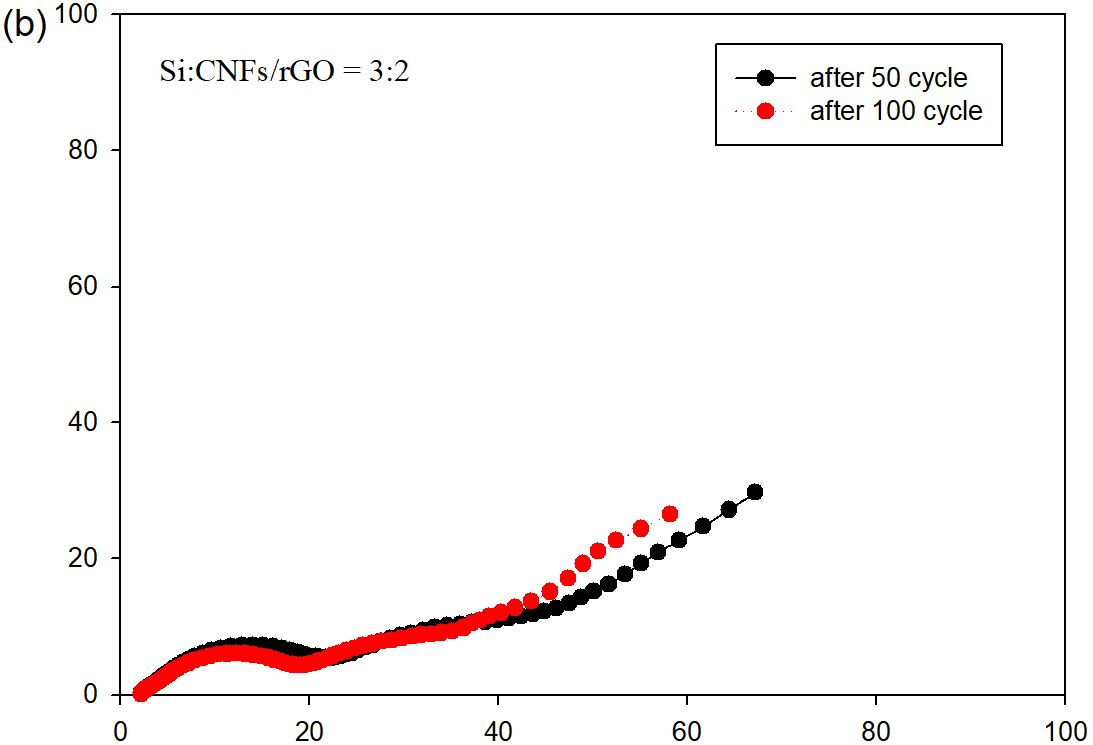

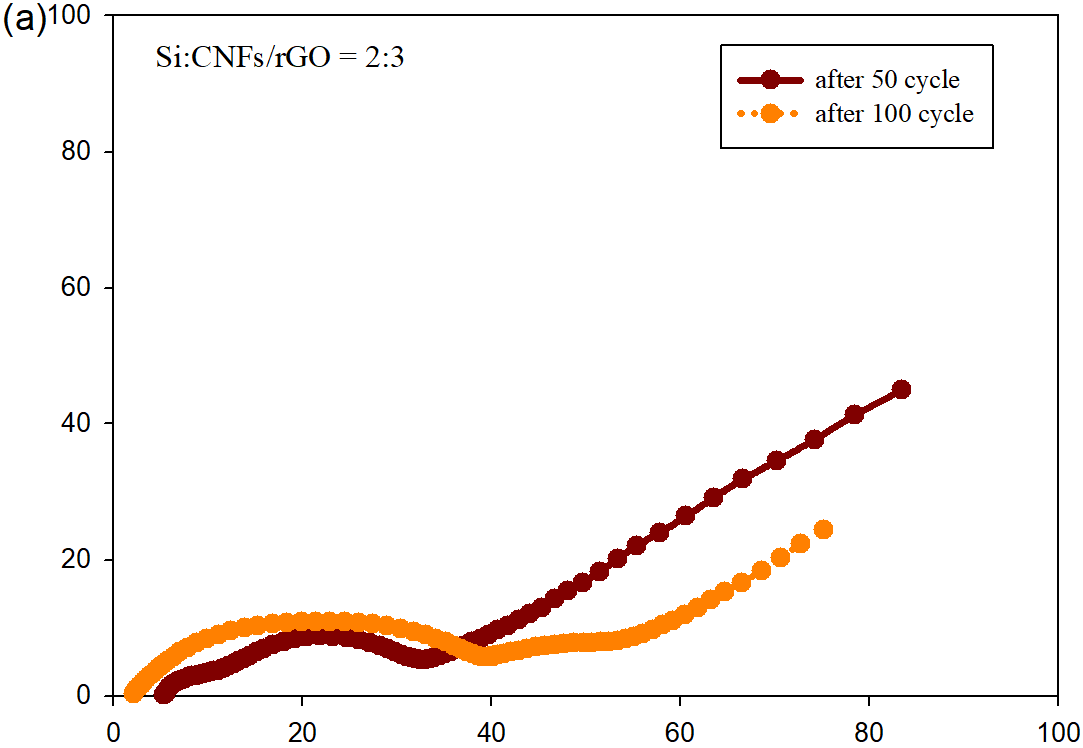


Z''/Ω

Z'/Ω

Z''/Ω

Z'/Ω


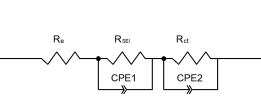

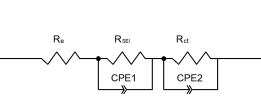


**Fig. S4.** Nyquist plots and electrochemical impedance spectra of the Si:CNF/rGO = 2:3 and Si:CNF/rGO = 3:2 anodes after 50 and 100 cycles.


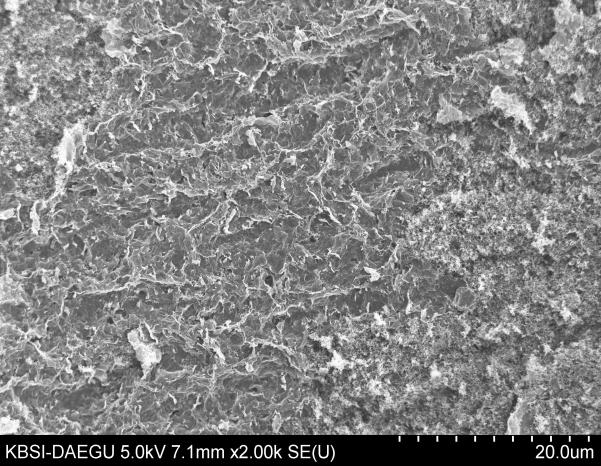


20μm


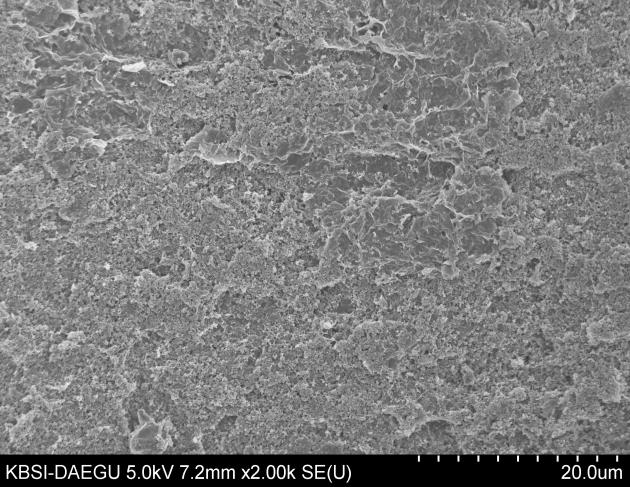


20μm


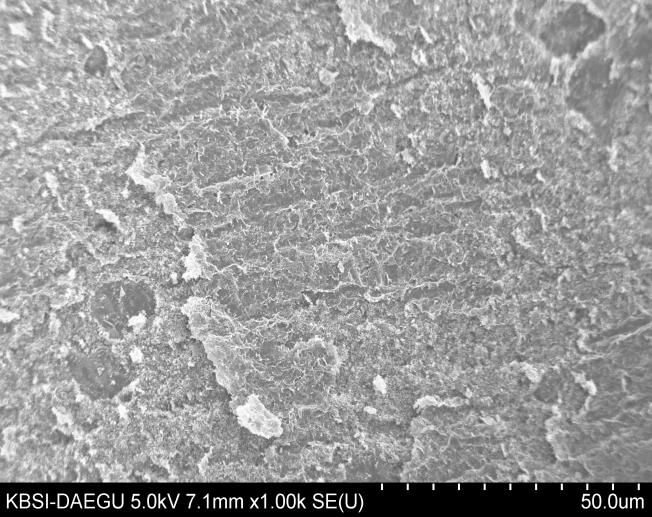


50μm


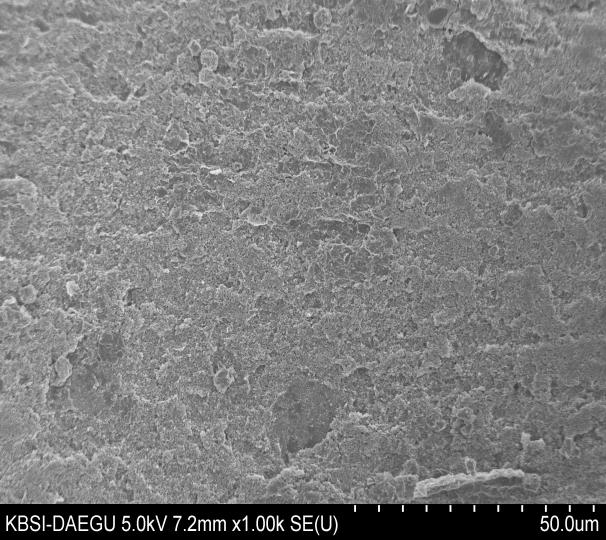


50μm


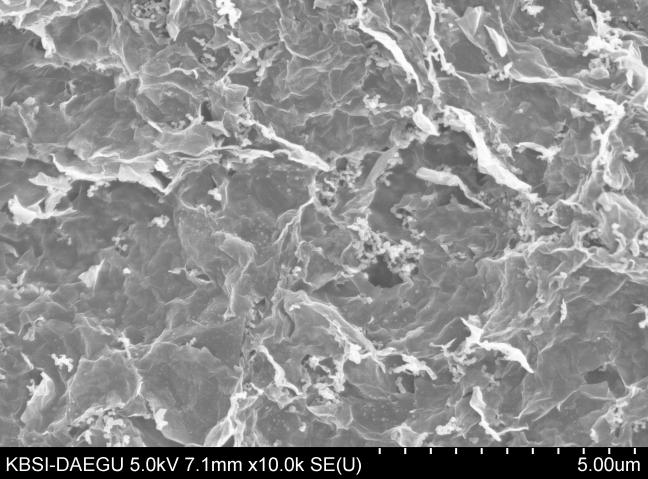


5μm


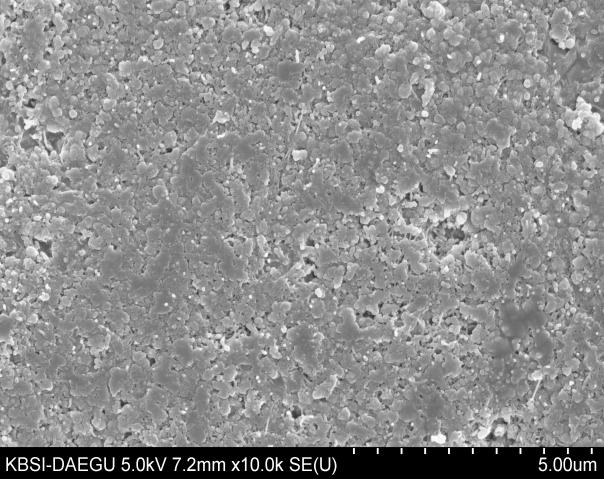


5μm

(a)

(f)

(e)

(d)

(c)

(b)

**Fig. S5** (a,b,c) SEM images of the surface of Si/CNF/rGO electrodes before the first cycle, (d,e,f) SEM images of the surface of Si/CNF/rGO electrodes after 100th cycles at a current density of 100mAg^-1^.

**Table S1.** EDX spectral results of the Si:CNF/rGO = 1:1, 3:2, and 2:3 composites.

| **Sample** | Silicon | | Carbon | | Oxygen | |
| --- | --- | --- | --- | --- | --- | --- |
|  | **Mass (%)** | Atom% | **Mass (%)** | Atom% | **Mass (%)** | Atom% |
| Si:CNF/rGO = 1:1 | 38.16 | 21.37 | 54.59 | 71.50 | 7.25 | 7.13 |
| Si:CNF/rGO = 3:2 | 48.94 | 30.08 | 41.37 | 59.46 | 9.69 | 10.46 |
| Si:CNF/rGO = 2:3 | 29.27 | 15.71 | 56.43 | 70.81 | 14.31 | 13.48 |

**Table S2.** Raman spectroscopy results of the GO, rGO, CNFs, Si/rGO, and Si/CNF/rGO samples (see Fig. 4(b)).

|  | ID/IG |
| --- | --- |
| Si/CNF/rGO | 1.02 |
| Si/rGO | 1.04 |
| CNFs | 0.99 |
| rGO | 0.96 |
| GO | 1.09 |

**Table S3.** Discharge capacity, coulomb efficiency, and capacity retention rate of the Si/rGO and Si:CNF/rGO = 1:1, 3:2, and 2:3 composite electrodes.

| Samples | Max. discharge  capacity  (mAh/g) | Discharge capacity (mAh/g) | | Coulomb efficiency (%) | | Capacity retention rate (%) | |
| --- | --- | --- | --- | --- | --- | --- | --- |
|  |  | **After 50 cycle** | **After 100 cycle** | **first cycle** | **100 cycle** | **After 50 cycle** | **After 100 cycle** |
| Si/rGO | 3,743.8 | 362.2 | 277.3 | 36.3 | 99.2 | 9.7 | 7.4 |
| Si:CNF/rGO = 1:1 | 3,634.9 | 1,064.1 | 964.7 | 26.0 | 93.8 | 29.3 | 26.5 |
| Si:CNF/rGO = 3:2 | 3,734.3 | 775.1 | 545.9 | 51.7 | 100.0 | 20.8 | 14.6 |
| Si:CNF/rGO = 2:3 | 2,339.1 | 647.5 | 462.9 | 28.8 | 100.0 | 27.7 | 19.8 |

**Table S4.** R_CT_ and R_SEI_ values calculated from the EIS spectra (see Fig. 7).

| Samples | R_CT_ (Ω) | R_SEI_ (Ω) | |
| --- | --- | --- | --- |
| Si/rGO | 546.8 | 58.2 |  |
| Si:CNF/rGO = 1:1 | 247.3 | 12.0 |  |
| Si:CNF/rGO = 2:3 | 372.0 | 36.7 |  |
| Si:CNF/rGO = 3:2 | 374.3 | 17.9 |  |
